# Supplementary material for: GATOR1 Mutations Impair PI3 Kinase-Dependent Growth Factor Signaling Regulation of mTORC1
Source: Int J Mol Sci. 2024 Feb 8;25(4):2068. doi: 10.3390/ijms25042068 (PMC10889792; doi:10.3390/ijms25042068)
Supplement: Supplementary file 1 [file ijms-25-02068-s001.zip › ijms-2859852-supplementary.pdf]

TABLE S1. Primers used for NPRL2 site-directed mutagenesis. BOLD indicates mutation. (*=stop)

NPRL2 variants	Primers	Sequence
34*	Forward	5'-GAGGATTTCATCTCG**T**GAGAACTGTTTGACAC-3'
	Reverse	5'-GTGTCAAACAGTTCTC**A**CGAGATGAAATCCTC-3'
L105P	Forward	5'-CATCGTTAAAAAGC**C**GGCCGGCTACTTGAC-3'
	Reverse	5'-GTCAAGTAGCCGGCC**G**GCTTTTTAACGATG-3'
T110S	Forward	5'-CGGCTACTTGA**G**CACGCTGGAGCTTGA-3'
	Reverse	5'-TCAAGCTCCAGCGTG**C**TCAAGTAGCCG-3'
D214H	Forward	5'-TCTCCGCTGAGGCA**C**ACGTGGAACTCAAC-3'
	Reverse	5'-GTTGAGTTCCACGT**G**TGCCTCAGCGGAGA

Table S2. List of antibodies used in this study.

Protein Target	Vendor	Catalog #
AKT	Cell Signaling Technology	9272
Beta-Actin	Cell Signaling Technology	3700
DEPDC5	Invitrogen	PA571618
FLAG	Sigma	F1804
GAPDH	Sigma	G9295
NPRL2	Santa Cruz Biotechnology, Inc.	sc-376986
NPRL3	Abcam	ab121346
p70 S6 Kinase	Cell Signaling Technology	9202
Phospho-p70 S6 Kinase (Thr389)	Cell Signaling Technology	9205
Phospho-AKT (Thr308)	Cell Signaling Technology	4056
Phospho-AKT (Ser473)	Cell Signaling Technology	9271
Phospho-S6 (Ser240/244)	Cell Signaling Technology	2215
Phospho-TSC2 (Thr1462)	Cell Signaling Technology	3617
RAGA	Cell Signaling Technology	4357
RHEB	Cell Signaling Technology	13879
RHEB-GTP	New East Biosciences	26910
S6	Cell Signaling Technology	2217
Sec13	Santa Cruz Biotechnology, Inc.	sc-514308
TSC2	Cell Signaling Technology	3612

SUPPLEMENTAL FIGURES:

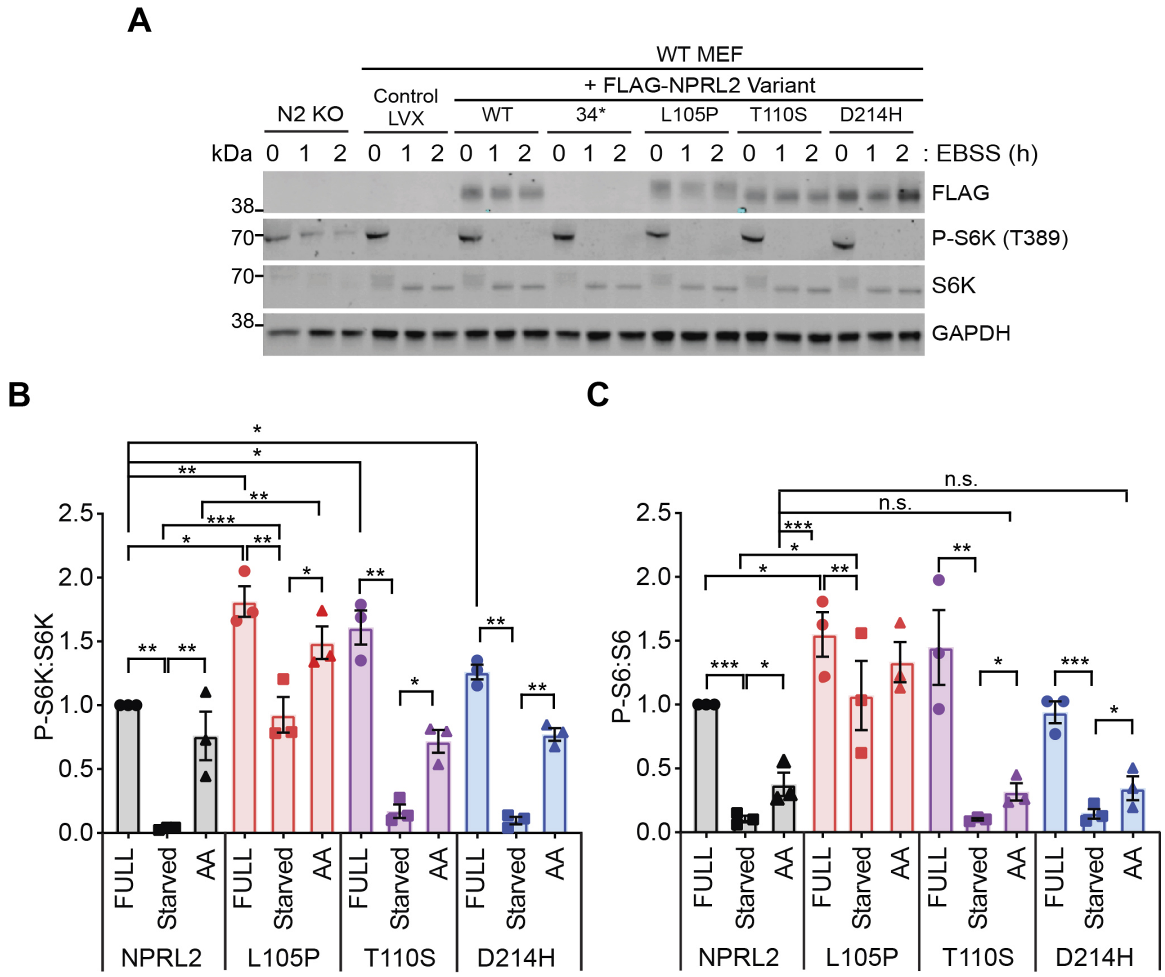

Figure S1. **(A)** NPRL2 KO MEF or WT MEF cells expressing either FLAG-NPRL2, FLAG-NPRL2(34*), FLAG-NPRL2(L105P), FLAG-NPRL2(T110S), or FLAG-NPRL2(D214H) were treated with EBSS for 1 or 2 hours. Protein extracts were analyzed by western blotting toward mTORC1 target P-S6K (T389), total S6K, and GAPDH. Quantification of the ratio of Figure 1C western blots toward **(B)** P-S6K (T389):S6K and **(C)** P-S6 (Ser240/244):S6 intensities normalized to β-actin. The ratio of each lane was normalized to β-actin and shown relative to the control (WT) in full medium. *$p < 0.05$, **$p < 0.01$, ***$p < 0.001$. n=3.

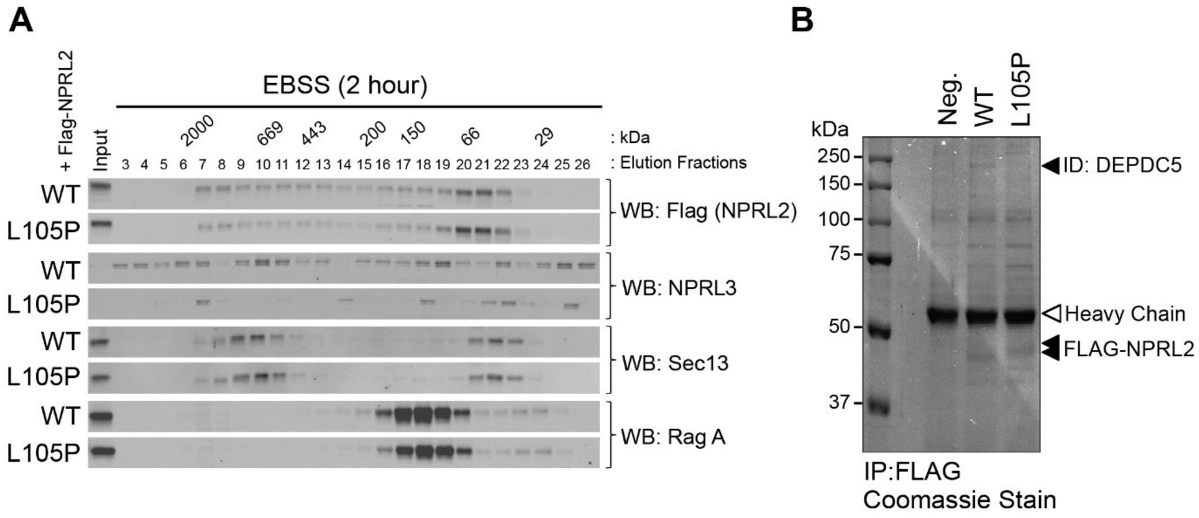

Figure S2. (A) Size exclusion chromatography was used to separate protein extracts from MEF cells expressing FLAG-NPRL2 and FLAG-NPRL2-L105P treated with EBSS for 2 hours and western blotting was used determine the distribution of FLAG with GATOR1 subunit NPRL3, GATOR2 subunit Sec13, and GATOR1 target RAGA. **(B)** Coomassie-stained SDS-PAGE gel of proteins from FLAG immunoprecipitation reactions using wild-type control MEF cells (Neg.) or NPRL2 KO cells stably expressing FLAG-NPRL2 or FLAG-NPRL2(L105P). Data represents n=3 independent experiments. DEPDC5 identification in FLAG-NPRL2 (WT lane) was made using mass spectrometry fingerprinting.

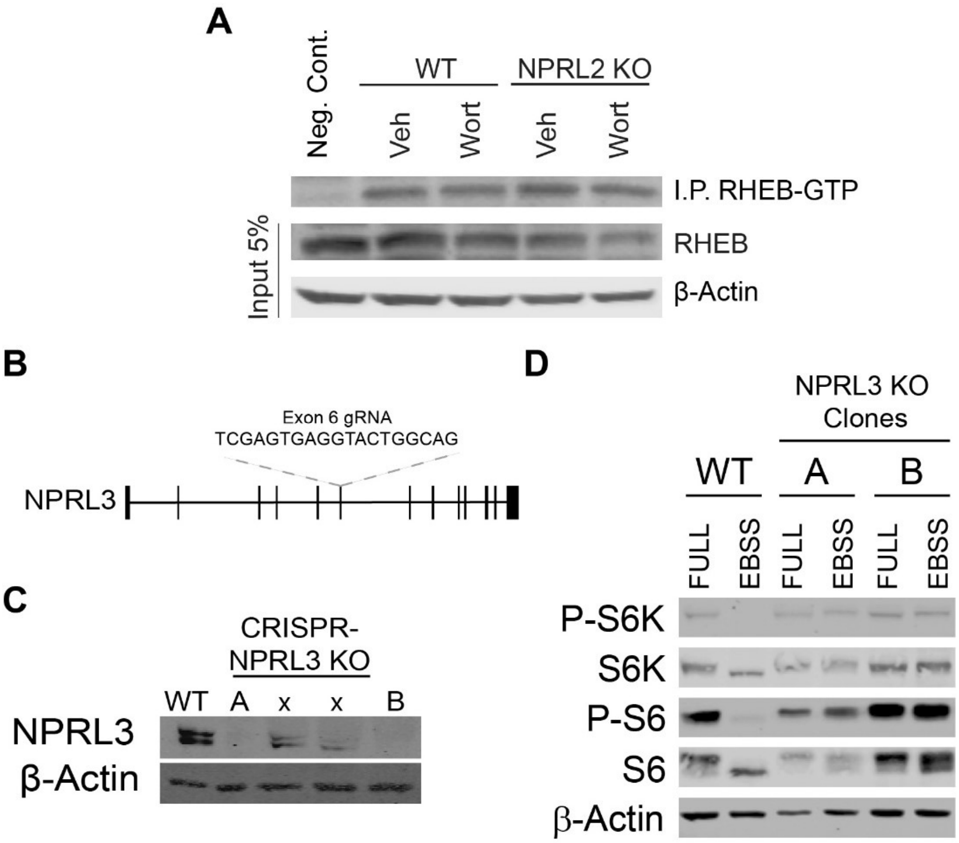

FIGURE S3. (A) GTP-binding state of RHEB from WT and NPRL2 KO cells treated with wortmannin for 1 hour. Immunoprecipitation was performed using antibodies targeting the RHEB-GTP before western blotting against RHEB-GTP, total RHEB and β-actin. Negative control lane did not include RHEB-GTP antibody in the precipitation reaction. **(B)** CRISPR-mediate NPRL3 knockout targeting scheme. **(C)** NPRL3 western blot analysis from WT and NPRL3 KO cell lines. **(D)** EBSS treatment of WT or NPRL3 KO cell-lines were cultured in either complete media or EBSS for 1 hour. Western blot analysis shows NPRL3 KO cells are unable to fully repress mTORC1 signal transduction toward downstream targets S6K and S6.
